# Correlation between invasion front and survival of patients with cutaneous melanomas^؆^

**DOI:** 10.1016/j.abd.2026.501409

**Published:** 2026-07-03

**Authors:** Vinícius Marinho Carvalho, Leonardo Emílio Sátiro Bezerra, Francisco Alves Moraes Neto, Juliana Polizel Ocanha-Xavier, Marcel Arakaki Asato, José Cândido Caldeira Xavier-Júnior

**Affiliations:** a Department of Pathology, Faculty of Medicine, Centro Universitário Católico Salesiano Auxilium, Araçatuba, SP, Brazil; b Hospital Amaral Carvalho, Jaú, SP, Brazil; c Department of Dermatology, Private Practice, Araçatuba, SP, Brazil; d Department of Pathology, Faculty of Medicine, Universidade Federal do Mato Grosso do Sul, Campo Grande, MS, Brazil; e Department of Dermatopathology, Instituto de Patologia de Araçatuba, Araçatuba, SP, Brazil; f Hospital Santa Casa de Araçatuba, Araçatuba, SP, Brazil

Dear Editor,

Tumor growth pattern, both macroscopic and microscopic, is considered a relevant prognostic factor in several tumors, especially among carcinomas.^1^ Evidence regarding tumors of the pancreas,^1^ oral cavity,^2^ liver,^3^ colon and rectum^4^ and thyroid,^5^ demonstrates that tumors with an infiltrative growth pattern exhibit more aggressive biological behavior with unfavorable clinical outcomes.^1^, ^2^, ^3^, ^4^, ^5^ A study conducted with 76 cases of melanoma found a higher frequency of characteristics associated with a worse prognosis, such as lymphovascular and perineural invasion, among cases with an infiltrative growth pattern.^6^

Given the evident need for investigation into the prognostic implications of the invasion front in cutaneous melanomas, this study aimed to evaluate the correlation between invasion front (invasive × expansive) and survival of patients with cutaneous melanoma staged pT2, pT3, or pT4,^7^ treated between 2005 and 2021 at an oncology center in the interior of Brazil, associated with clinical, epidemiological, and anatomopathological factors. Classification was performed according to Fig. 1, Fig. 2, in which tumors whose invasive component showed growth with blunt borders were considered "expansive," while tumors whose invasive component showed an acute angle architecture / isolated cells – or in small groups – were considered "infiltrative."

**Fig. 1:**
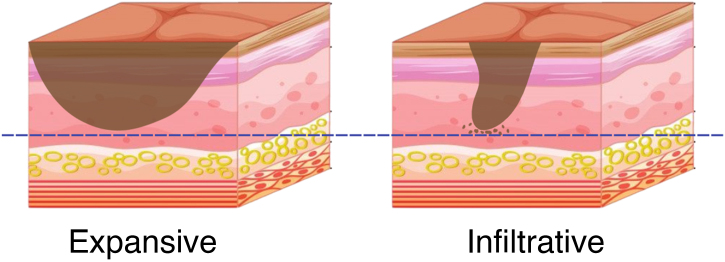
Comparative illustration representing tumor growth and infiltration patterns used in the classification of each type of invasion front, considering similar Breslow thickness (blue line).

**Fig. 2:**
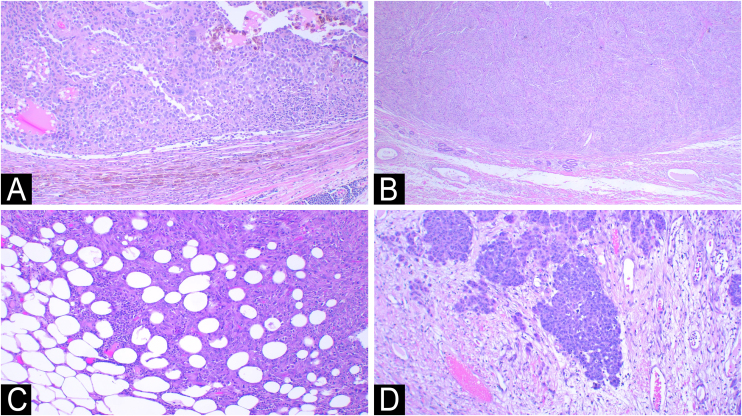
Visual representation of examples of “expansive” and “infiltrative” invasion fronts; (A and B) two examples of an expansive invasion front pattern in which the deeper edge of the lesion is more organized and well-defined, with “crescent-shaped” growth (Hematoxylin & eosin; ×40); (C and D) two examples of infiltrative invasion front patterns where the deepest portion of the lesion presents more irregularly, infiltrating the adipose tissue in a "honeycomb"-like pattern (image C, Hematoxylin & eosin ×40) or with an invasion front exhibiting acute-angle /triangular growth (image D, Hematoxylin & eosin, ×100).

Microscopic analysis was performed independently by two dermatopathologists with experience in the area. For discordant cases, a consensus discussion was held. Regarding the outcome, patients were divided into two groups: death due to melanoma *versus* survivors plus deaths due to other causes. Initially, a descriptive analysis was performed, calculating the mean, standard deviation, minimum and maximum values, and median for quantitative variables, as well as absolute and relative frequencies for categorical variables. The Chi-square test was used to assess the association between the invasion front and histological subtype and sentinel lymph node result. Considering survival in months, Kaplan-Meier curves were constructed, followed by the application of the log-rank test for variables of interest. Correlation between the invasion front and Breslow was evaluated by fitting the Cox regression model. In all tests, a significance level of 5% or the corresponding p-value was adopted. All analyses were performed using the SAS for Windows program, version 9.4.

Melanoma samples from 232 patients with a mean age of 63 years (14–87) were analyzed, comprising 112 (48.3%) women and 120 (51.7%) men. The mean Breslow thickness was 3.7 mm (1.1 mm – 25.7 mm). Regarding sentinel lymph nodes, 124 (53.5%) were negative and 108 (46.5%) were positive. Of these cases, 82 (35.3%) were of the superficial spreading subtype, 63 (27.2%) were of the acral type, 83 (35.8%) were of the nodular subtype, and the remaining four (1.7%) corresponded to other less common subtypes. Regarding the invasion front, 49 (21.1%) cases were classified as exhibiting an infiltrative front and 183 (78.9%) an expansive front. There was no association between the invasion front and histological subtype (p = 0.4394) nor with the sentinel lymph node result (p = 0.7013). Considering patients who died from melanoma *versus* other patients (alive with cancer, alive disease-free, or who died from other causes), survival estimated by the Kaplan-Meier curve stratified by invasion front did not reveal a significant result (p = 0.1354 log-rank test), as shown in Fig. 3. Adjusting a Cox model, considering survival time as the response variable, and Breslow and invasion front as explanatory variables, only Breslow showed a significant effect with HR = 1.13, 95% CI (1.09‒1.17; p < 0.0001).

**Fig. 3:**
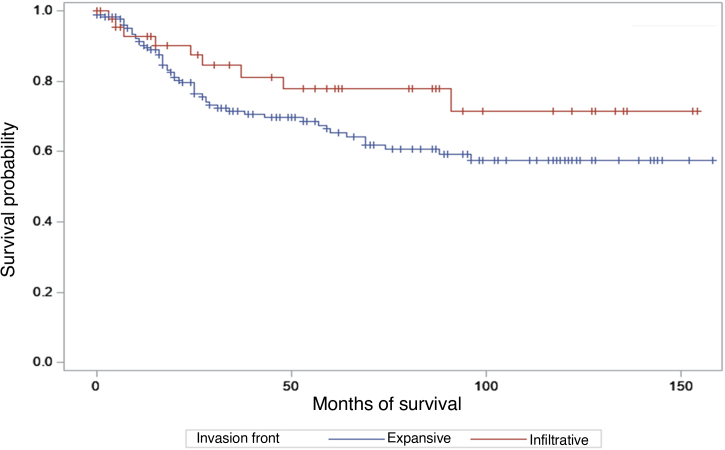
Kaplan-Meier curve comparing the invasion front with survival.

Although invasion front shows a strong correlation with survival in other tumor types,^1^, ^2^, ^3^, ^4^, ^5^ data from the present study did not identify an association between invasion front and survival in cases of invasive melanoma in the studied population. Furthermore, for melanomas, the search for histological biomarkers related to prognosis is emerging, such as the number of cells arranged individually – or in small groups of four cells or less – at the deeper edge of the tumor (tumor budding),^8^ or even the density of cells present in the invasive component of the lesion (Breslow density);^9^ a histological characteristic with the capacity to determine prognosis beyond the depth or shape of the lesion.^9^ Tumor budding, which has also shown a possible prognostic role in recent studies,^8^ although exhibiting some similarity to the concept of invasion front, cannot be considered equivalent since not every tumor with an infiltrative invasion front will have a high tumor budding and even tumors with an expansive invasion front may present soft tissue infiltration with clusters consisting of a small number of cells.

To illustrate these concepts comparatively, while the cases relating to images 2A and 2B show an expansive invasion front, they also exhibit a high Breslow density and low tumor budding. As for images 2C and 2D, which represent the infiltrative invasion front, the case relating to image 2C would have a high Breslow density and tumor budding, while the case relating to image 2D would have a low Breslow density and an intermediate tumor budding. This occurs because Breslow density considers the area (in percentage) affected by tumor cells at the Breslow measurement site without considering the shape/pattern of infiltration^9^ while budding considers the number of cells in clusters located in the deeper portion of the neoplasm.^8^ Thus, it should be emphasized that invasion front,^1^, ^2^, ^3^, ^4^, ^5^, ^6^ budding^8^ and Breslow density^9^ are different concepts, even though they may sometimes appear correlated, and it is not possible to extrapolate data from studies that address these different concepts separately. Therefore, although no correlation was observed between survival of patients with cutaneous melanomas and the invasion front pattern in the group of studied patients, further studies, which also evaluate surgical treatments and systemic therapies performed, may be necessary for a definitive conclusion regarding the applicability of this biomarker for patients with cutaneous melanomas.

## ORCID ID

Vinícius Marinho Carvalho: 0009-0006-3633-9745

Leonardo Emílio Sátiro Bezerra: 0009-0007-4738-2383

Francisco Alves Moraes Neto: 0009-0001-7820-8106

Juliana Polizel Ocanha-Xavier: 0000-0002-1200-3730

Marcel Arakaki Asato: 0000-0002-6050-5292

## Statements

The study was approved by the Research Ethics Committee of Hospital Amaral Carvalho (CAAE 52618721.0.3001.5434).

## Financial support

None declared.

## Authors' contributions

Vinícius Marinho Carvalho: Collection of data; drafting and editing of the manuscript; critical review of the literature.

Leonardo Emílio Sátiro Bezerra: Collection of data; drafting and editing of the manuscript; critical review of the literature.

Francisco Alves Moraes Neto: Original diagnostic microscopic analysis of cases.

Juliana Polizel Ocanha-Xavier: Critical review of important intellectual content.

Marcel Arakaki Asato: Collection and interpretation of data; critical review of the literature.

José Cândido Caldeira Xavier-Júnior: Design and planning of the study; collection of data; analysis and interpretation of data; statistical analysis; critical review of important intellectual content; collection, analysis and interpretation of data; effective participation in research orientation; critical review of the literature; approval of the final version of the manuscript.

## Research data availability

The entire dataset supporting the results of this study was published in this article.

## Conflicts of interest

None declared.
